# Characteristics of admissions to a Hospitalization Unit in a rural population during the COVID-19 pandemic

**DOI:** 10.1192/j.eurpsy.2023.508

**Published:** 2023-07-19

**Authors:** G. M. Chauca Chauca, M. R. Galán Armenteros, L. Carrión Expósito

**Affiliations:** Hospital Infanta Margarita, córdoba, Spain

## Abstract

**Introduction:**

On January 7, 2020, the Chinese authorities identified a new type of virus from the Coronaviridae family as the causative agent of an outbreak of pneumonia of unknown etiology, which has been named SARS-CoV-2. The disease caused by this new virus has been named by international consensus COVID-19. The WHO recognized it as a global pandemic on March 11, 2020. The Government of Spain declared the State of Alarm on March 13.

Hospitals have had to reorganize their consultation areas and emergency rooms to carry out security measures and prioritize the care of patients with COVID-19. All this has had repercussions on the closure of Psychiatric Day Hospitals and outpatient consultations, carrying out fundamentally telephone or telematic follow-up.

**Objectives:**

The objective of the study was to analyze the characteristics of admission during the year after the 2020 pandemic compared to the similar period in 2019.

**Methods:**

An observational study of retrospective characteristics of patients admitted to a hospitalization unit during the year 2020 after the pandemic will be carried out compared to the year 2019 of the same period. Demographic and clinical variables are included in the study.

**Results:**

During the period after the 2020 pandemic, a total of 135 patients were admitted, with a mean age of 42.8 years, 65 of them women and 70 men. With a diagnosis at discharge of 48% of Psychotic Disorder, 17% of Bipolar Disorder, 11.1% of other affective disorders (T. depressive, adaptive, dysthymia) 14% of Personality Disorder, and 17% of others. Compared to the same period of the previous year, the number of admissions decreased by 98 patients (42%), including the severity of the clinic, with 36% of the total admissions being psychotic disorders.

**Conclusions:**

It can be concluded that the number of hospitalizations has decreased due to the patients’ fear of being admitted and therefore being subject to infection, and the higher percentage of psychotic and affective pathology because these patients are more serious, making home containment impossible.

**Disclosure of Interest:**

None Declared

